# Cyclin A1 in Oocytes Prevents Chromosome Segregation And Anaphase Entry

**DOI:** 10.1038/s41598-020-64418-1

**Published:** 2020-05-04

**Authors:** Lenka Radonova, Tereza Pauerova, Denisa Jansova, Jitka Danadova, Michal Skultety, Michal Kubelka, Martin Anger

**Affiliations:** 1 Central European Institute of Technology, Department of Genetics and Reproduction, Veterinary Research Institute, Brno, Czech Republic; 2 Institute of Animal Physiology and Genetics, Czech Academy of Sciences, Libechov, Czech Republic

**Keywords:** Oogenesis, Meiosis, Development

## Abstract

In several species, including Xenopus, mouse and human, two members of cyclin A family were identified. Cyclin A2, which is ubiquitously expressed in dividing cells and plays role in DNA replication, entry into mitosis and spindle assembly, and cyclin A1, whose function is less clear and which is expressed in spermatocytes, leukemia cells and in postmitotic multiciliated cells. Deletion of the gene showed that cyclin A1 is essential for male meiosis, but nonessential for female meiosis. Our results revealed, that the cyclin A1 is not only dispensable in oocytes, we show here that its expression is in fact undesirable in these cells. Our data demonstrate that the APC/C and proteasome in oocytes are unable to target sufficiently cyclin A1 before anaphase, which leads into anaphase arrest and direct inhibition of separase. The cyclin A1-induced cell cycle arrest is oocyte-specific and the presence of cyclin A1 in early embryos has no effect on cell cycle progression or chromosome division. Cyclin A1 is therefore not only an important cell cycle regulator with biased expression in germline, being essential for male and damaging for female meiosis, its persistent expression during anaphase in oocytes shows fundamental differences between APC/C function in oocytes and in early embryos.

## Introduction

Cyclins are proteins which, together with cyclin dependent kinases (Cdks), control cell cycle progression^1,2^. Several cyclins are expressed as structurally and functionally different variants, for example cyclin B in mammals is known as B1, B2 and B3^2^. Similarly, two forms of cyclin A were identified, first in Xenopus^3^, and then also in mouse^4^ and human^5^. The canonical cyclin A, named cyclin A2, is essential for DNA replication as well as for mitosis and therefore it is ubiquitously expressed in almost all cell types. The expression of the second A cyclin family member, cyclin A1, is limited to the germ line cells^4^, certain leukemia cells^6^ and surprisingly also to the brain cells^7^. Although our knowledge about the function of cyclin A1 is limited, it was shown that similarly to cyclin A2, it forms complexes with Cdk2 as well as with Cdk1^5,8^. It was also shown that cyclin A1 can bind to various DNA repair proteins^9^, indicating that it might be involved in DNA double-strand break repair^10^. And recently cyclin A- Cdk2 complex was implicated in ciliogenesis in postmitotic multiciliated cells^11^.

Gene targeting studies revealed that cyclin A1 gene (CCNA1) is in mouse essential for completion of male meiosis, but it is not required for female meiosis^12^. The expression of CCNA1 in spermatocytes is controlled by the sequence motif within the promoter of the gene^13^ and transcription from both alleles is required in order to reach sufficient concentration of the protein within the cell^7^. During spermatogenesis, cyclin A1 is detectable in stages IX-XII, up to the meiosis I division, whereas in meiosis II, the protein was not detected^14,15^. This is consistent with the phenotype resulting from the deletion of the CCNA1, which is causing arrest at the diplotene stage of meiosis I^12^. Interestingly, the deletion of Cdk2 arrests male meiosis earlier, in late pachytene stage^16^. It was therefore hypothesized that cyclin A1 might have a role, which does not require Cdk2 or that it might form an active complex with another Cdk^17^. It was also suggested that cyclin A1 - Cdk2 complex might play a role in activation of CDC25 during the first meiotic division in spermatocytes^18^.

In this study we focused on cyclin A1 in oocyte meiosis. It was previously shown that this molecule is not essential for successful completion of female meiosis, but it seems that there is no consensus whether it is even expressed in mouse oocytes^19,20^. We analyzed the phenotype after expression of cyclin A1 in fully-grown mouse GV oocytes. These cells are at similar stage of meiosis as spermatocytes arrested by cyclin A1 deletion. Surprisingly, the expression of cyclin A1 in GV oocytes caused failure to complete meiosis. Our experiments revealed that the APC/C and proteasome are in oocytes unable to remove completely cyclin A1 before anaphase, which leads into failure to extrude the polar body and to direct inhibition of separase. To our knowledge this is the first example of an important cell cycle regulator exhibiting such strong bias between male and female meiosis. Our results showed that cyclin A1 has deleterious effect on female meiosis.

## Results

### Cyclin A1 expression in oocytes prevents chromosome segregation and anaphase entry

**Figure 1:**
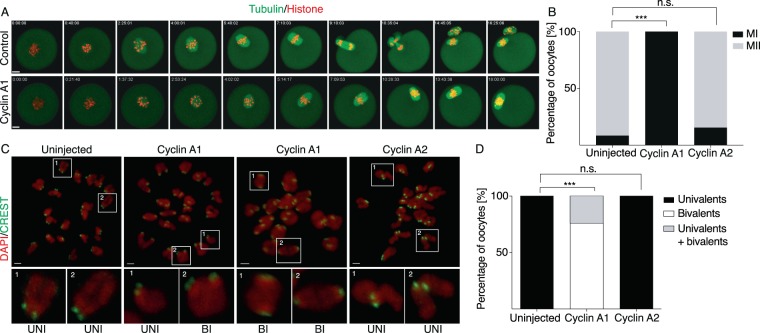
Expression of cyclin A1 in mouse GV oocytes blocks polar body extrusion and conversion of bivalents into univalent. (**A**) Frames from live cell imaging experiment of oocytes microinjected with cRNAs encoding tubulin (green) and histone (red) fused to fluorescent proteins, oocyte in lower panel was also co-injected with cRNA encoding cyclin A1. Scale bar represents 10 μm. (**B**) Scoring of maturation in control – uninjected oocytes (n = 84), oocytes injected with cyclin A1 (n = 44) or cyclin A2 (n = 52) cRNAs. The chart shows percentage of oocytes in meiosis I and meiosis II (control 92% MII; cyclin A1 0% MII; cyclin A2 85% MII) after overnight maturation in each category. The data were obtained in three independent experiments. The difference between control oocytes and oocytes injected with cyclin A1 cRNA was statistically significant (α < 0.05; ***P < 0.0001). The difference between control oocytes and oocytes injected with cyclin A2 cRNA was not statistically significant (α < 0.05; P = 0.2021). (**C**) Confocal microscopy images showing DNA stained by DAPI (red) and kinetochores stained by CREST antibody (green) in control – uninjected oocytes (left panel), oocytes injected with cyclin A1 cRNA (two central panels) and oocytes injected with cyclin A2. Scale bar represents 2 μm. (**D**) Scoring of chromosomes status after overnight maturation of control – uninjected oocytes (n = 36), oocytes injected with cyclin A1 cRNA (n = 37) and oocytes injected with cyclin A2 cRNA (n = 43). The percentage of oocytes carrying univalents (control 100%, cyclin A1 0%, cyclin A2 100%), bivalents (control 0%, cyclin A1 76%, cyclin A2 0%) or both (control 0%, cyclin A1 24%, cyclin A2 0%) after overnight meiotic maturation is indicated. The data were obtained in three independent experiments. The difference between control oocytes and oocytes injected with cyclin A1 cRNA was statistically significant (α < 0.05; ***P < 0.0001). The difference between control oocytes and oocytes injected with cyclin A2 cRNA was not statistically significant (α < 0.05; P > 0.9999).

Gene targeting^12^ showed that cyclin A1 is not essential for oocyte development and meiotic division in mouse. However, from published literature^19,20^ it is not clear whether this protein is present in oocytes at all. In order to assess its effect on meiotic division, GV oocytes were microinjected with cRNAs encoding tagged histone and tubulin and untagged cyclin A1, and the first meiotic division was monitored using confocal time lapse imaging (Fig. 1A). Surprisingly, expression of cyclin A1 arrested cells in meiosis I. To assess quantitatively the frequency of meiosis I arrest upon cyclin A1 expression, we performed another set of experiments, in which we compared uninjected oocytes with oocytes injected with cRNAs encoding untagged cyclins A1 or A2 (Fig. 1B). After microinjection at the GV stage, all cells were matured in the incubator and the rate of the polar body extrusion (PBE) was evaluated after 20 hours. Our results showed that whereas 92% of uninjected cells and 85% of cells injected with cyclin A2 underwent PBE, 100% of cells injected with cyclin A1 were arrested in meiosis I. To analyze whether in oocytes injected with cyclin A1 bivalent chromosomes were converted into univalents, cells from the previous experiment were stained by DAPI and CREST and the configuration of chromosomes was assessed (Fig. 1C). The results (Fig. 1D), showed that all chromosomes in uninjected or cyclin A2 injected MII cells were univalents. In contrast to this, 76% of oocytes injected with cyclin A1 contained only bivalents and 24% contained bivalents together with univalents. In this group there were no oocytes containing only univalents. This suggested that the meiosis I arrest, observed in oocytes injected with cyclin A1, prevented also chromosome division.

### Cyclin A1 mRNA levels in GV oocytes are extremely low

It was obvious from our initial experiments that cyclin A1 in oocytes has a deleterious effect on polar body extrusion and chromosome segregation. We therefore aimed to assess levels of cyclin A1 mRNA in GV oocytes using qPCR. Total RNA was isolated from mouse GV oocytes and from suspension of spermatogenic cells. After reverse transcription, target sequences within cyclins A1, A2 and B1 were amplified using TaqMan probes. Because it would be difficult to compare directly the mRNA expression between oocytes and spermatocytes, the ratios between expression of cyclin A1 and A2 and B1 cyclins, which are both essential for meiosis^21,22^, were established for oocytes and spermatocytes separately (Tab. 1). The difference between the threshold values (Ct) showed, that in both oocytes and spermatocytes the mRNA levels of cyclin A1 were lower than mRNAs of cyclins A2 and B1. During exponential phase of qPCR, the amount of DNA doubles every cycle. Therefore, in spermatocytes, the expression of cyclin A2 mRNA was about 13 times and cyclin B1 about 18 times higher than the cyclin A1 mRNA. In oocytes this ratio was dramatically higher, 119 for cyclin A2 and 162 for cyclin B1. Also, the average threshold cycle of cyclin A1 in oocytes was 38, which indicated, that this mRNA was at the border of detection. Such low expression of cyclin A1 is consistent with the previous report showing that cyclin A1 mRNA was undetectable in the ovary by *in situ* hybridization^20^.

### Oocytes are unable to efficiently target cyclin A1 for degradation before anaphase

**Figure 2:**
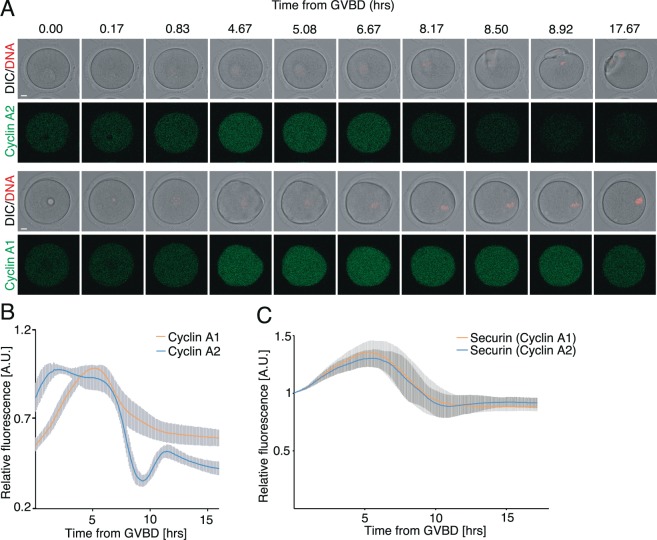
Oocytes are unable to efficiently target cyclin A1 for degradation. (**A**) Frames from live cell imaging experiment of oocytes co-injected with cRNAs encoding histone (red) and either cyclin A2 or cyclin A1 (green) fused to fluorescent proteins. Scale bar represents 10 μm. (**B**) Profiles of fluorescent signal of cyclin A1 (orange, n = 12) and cyclin A2 (blue, n = 7) during meiotic maturation. The curves represent average curve for each construct and the standard deviation error bars are shown. The signal in each cell was normalized to the frame closest to the disassembly of the nuclear membrane (GVBD). The data were obtained from two independent experiments. (**C**) Profiles of fluorescent signal of securin fused to fluorescent protein in oocytes injected by cyclin A1 (orange, n = 14) or cyclin A2 (blue, n = 10) cRNAs during meiotic maturation. The curves represent average expression curve for all cells in the group and the standard deviation error bars are shown. The signal in each cell was normalized to the frame closest to GVBD. The data were obtained from two independent experiments.

**Figure 3:**
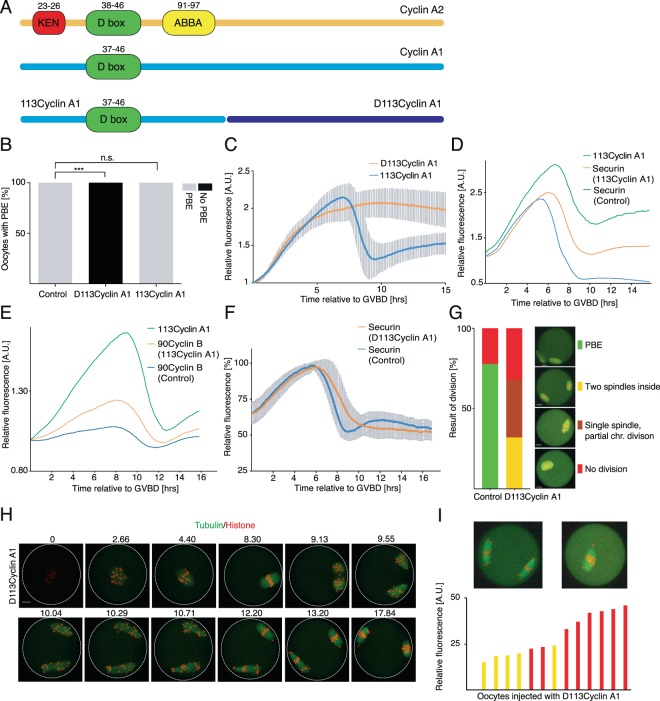
Fragment of cyclin A1 which contains D-box is not efficiently targeted for degradation and alters dynamic of degradation of other APC substrates. (**A**) Schematic comparison of cyclin A1 and cyclin A2 and the relative positions of sequence motives (D-box, KEN box, ABBA motive) related to the ubiquitination and degradation of the molecule on proteasome. (**B**) Scoring of PBE in control oocytes (n = 8) and oocytes injected with either 113cyclin A1 (n = 12) or D113cyclin A1 (n = 12) cRNAs. Grey bars represent oocytes with underwent PBE (control 100%, 113cyclin A1 100% and D113cyclin A1 0%), black bars represent oocytes arrested in meiosis I. The data were obtained from two independent experiments. The difference between control oocytes and oocytes injected with D113cyclin A1 cRNA was statistically significant (α < 0.05; ***P < 0.0001). The difference between control oocytes and oocytes injected with 113cyclin A1 cRNA was not statistically significant (α < 0.05; P > 0.9999). (**C**) Relative expression curves of 113cyclin A1 (blue, n = 12) and D113cyclin A1 (orange, n = 12) during oocyte meiosis. GV oocytes were injected with histone and cyclin A1 cRNAs fragments fused to fluorescent proteins. The fluorescence was measured overnight using confocal time lapse microscopy. Signal in each cell was normalized to the level at GVBD and the average curves are shown together with standard deviation values at each time point. The data were obtained from two independent experiments. (**D**) Relative expression curves of securin in control cells (blue, n = 11), of securin in cells co-injected with cRNA encoding 113cyclin A1 (orange, n = 17) and relative expression curve of 113cyclin A1 itself (green, n = 17). The fluorescence was measured overnight using confocal time lapse microscopy, the signal in each cell was normalized to the level at GVBD. The average curves are shown for each time point. The data were obtained from two independent experiments. (**E**) Relative expression curves of 90cyclin B1 in control cells (blue, n = 16), and in cells co-injected with cRNAs encoding 90cyclin B1 and 113cyclin A1 (orange, n = 14) and relative expression curve of 113cyclin A1 itself (green, n = 14). The fluorescence was measured overnight using confocal time lapse microscopy, the signal in each cell was normalized to the level at GVBD. The average curves are shown for each time point. The data were obtained from two independent experiments. (**F**) Relative expression curves of securin in control cells (blue, n = 14) and in cells co-injected with cRNAs encoding securin and D113cyclin A1 (orange, n = 25). The fluorescence was measured overnight using confocal time lapse microscopy, the signal in each cell was normalized to the level at GVBD. The average curves, together with standard deviations are shown for each time point. The data were obtained in three independent experiments. (**G**) Scoring of spindle division phenotypes in oocytes injected with histone and tubulin cRNAs fused to fluorescent proteins. The frequency of each phenotype is indicated for control cells (n = 18; PBE 78%, no division 22%) and for cells injected with D113cyclin A1 cRNA (n = 25; two spindles inside 32%; internal spindle and partial chr. division 36%; no division 32%). The data were obtained in three independent experiments. Scale bar represents 10 μm. (**H**) Frames from time lapse movie of oocyte co-injected with cRNAs encoding histone, tubulin and D113cyclin A1. Scale bar represents 10 μm. (**I**) Chart demonstrating a dependency of expression levels of D113cyclin A1 on chromosomes and spindle division. GV oocytes were injected with cRNAs encoding histone and tubulin fused to fluorescent proteins and with various amount of D113cyclin A1 cRNA diluted in water containing fluorescently labelled dextran, allowing to quantify the amount of D113cyclin A1 in each individual cell. Presented are data from one experiment.

Similarly to the somatic cells, entry into anaphase requires in oocytes inhibition of Cdk activity^23^. This is achieved by targeting cyclins carrying D-box for degradation by APC/C^24^. Meiosis I arrest, caused by cyclin A1 expression, resembled the phenotype after expression of cyclin B1 without the destruction box^25^. Cyclin A1, similarly to cyclin A2, has D-box located in the N-terminal part (Supplemental Fig. 1). In order to test whether cyclin A1 is targeted during meiosis I, GV oocytes were microinjected with cRNAs encoding tagged histone H2B and tagged cyclin A1 or A2. The results showed that although the expression levels of both cyclins decreased towards anaphase, only in case of cyclin A2 the signal almost completely disappeared at the transition between meiosis I and meiosis II, whereas the signal of cyclin A1 remained high (Fig. 2A,B). We further tested whether oocytes microinjected with cyclin A1 were able to target securin, which is another APC/C substrate^26^. Cells were microinjected with cRNAs of tagged securin and untagged cyclin A1 or A2. The results showed (Fig. 2C) that securin signal disappeared before anaphase similarly in both groups, indicating that the activity of APC/C and proteasome was not significantly perturbed by cyclin A1. It indicated that the inability to target microinjected cyclin A1 might be related to the protein itself. In order to confirm this, we prepared two constructs containing N-terminal and C-terminal fragments of cyclin A1 (Fig. 3A). Construct 113cyclin A1 contained first 113 N-terminal amino acids, including also D-box at position 37–46^27^ and the construct D113cyclin A1 contained C-terminal part of cyclin A1, starting with amino acid 114. The D113cyclin A1 thus lacked the D-box and contained the Cdk binding motive mapped to amino acids 439–452 within the C-terminal part of cyclin A2^28,29^. GV oocytes were microinjected with cRNAs encoding histone H2B and either 113cyclin A1 or D113cyclin A1, all tagged by fluorescent proteins. The control group was injected with cRNA of tagged histone H2B only. First, meiosis I division was assessed by scoring PBE (Fig. 3B). This showed that 100% of control oocytes as well as 100% of oocytes injected with 113cyclin A1 underwent PBE. In contrast to this, none of the oocytes injected with D113cyclin A1 extruded PB. The fluorescence signal of tagged 113cyclin A1 and D113cyclin A1 showed that only the fragment containing D-box was targeted for degradation, whereas the C-terminal fragment was stabilized (Fig. 3C). To analyze whether the targeting of other APC/C substrates is unperturbed by 113cyclin A1, we tested securin (Fig. 3D) and fragment of cyclin B1 containing the first 90 N-terminal amino acids (Fig. 3E). Our results showed that although the expression of 113cyclin A1 altered the dynamics of destruction of securin as well as cyclin B1 fragment, most of the oocytes underwent PBE (data not shown). It is worth mentioning that since 113cyclin A1 lacks the Cdk binding domain, the effect which this fragment has on destruction of other APC/C substrates does not require Cdk activity. Importantly though, experiments with 113cyclin A1 (Fig. 3C–E) demonstrated that its residual signal at the time of anaphase was always higher than at the GVBD, which was not the case of other APC/C substrates - securin (Figs. 2C and 3D), cyclin A2 (Fig. 2B) or the fragment of cyclin B1 (Fig. 3E), in which the level of signal at anaphase was always lower than at the GVBD. This demonstrates that the N-terminal fragment of cyclin A1, which contains D-box, was ineffectively targeted by APC/C and proteasome, similarly to the full-length cyclin A1. It was shown previously that the destruction of other APC/C substrates is in general not perturbed by expression of stable versions of cyclins lacking D-box^30^. We therefore tested the effect of D113cyclin A1 on securin targeting (Fig. 3F). Our results showed that the C-terminal fragment of cyclin A1 only moderately altered the dynamics of securin destruction (Fig. 2C).

Since in experiments shown in Fig. 3F, the cells were coinjected with D113cyclin A1 together with cRNAs of tagged histone H2B and tubulin, the behavior of the spindle and chromosomes was monitored simultaneously. We observed unusual behavior of the spindle, involving a division of the spindle and chromosomes inside the cell without PBE (Fig. 3G,H). Scoring of this phenotype showed that 32% of cells injected with D113cyclin A1 divided both spindle and chromosomes and 36% only partially chromosomes. In order to assess whether the variability of phenotype could be caused by different levels of D113cyclin A1, oocytes were injected with cRNAs encoding histone H2B, tubulin and D113cyclin A1, all fused to fluorescent proteins and also with fluorescently labelled dextran. The dextran signal allowed quantification of the injected D113cyclin A1 cRNA independently on transcription. The results (Fig. 3I) showed that the low levels of injected D113cyclin A1 were associated with spindle division and with increasing concentration the spindle division was blocked and only partial division of the chromosomes was observed.

### Separase is the target of cyclin A1 in oocyte meiosis I

**Figure 4:**
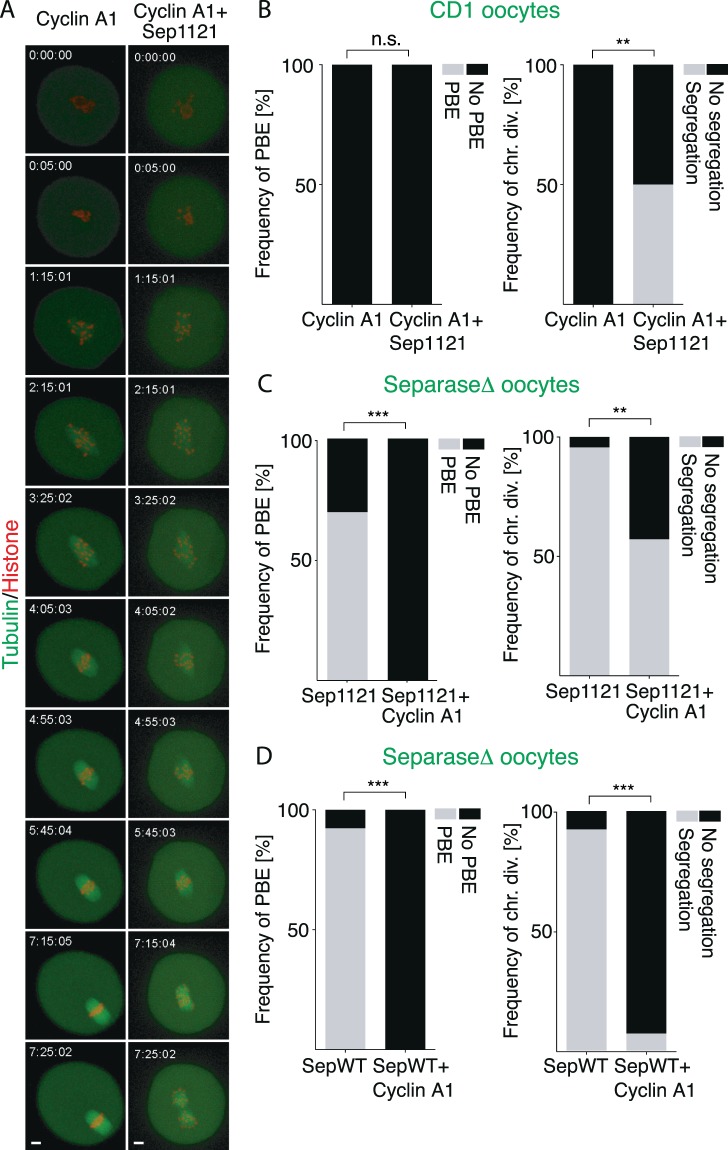
Cyclin A1 directly inhibits activity of separase. (**A**) Frames from confocal live cell imaging experiment of CD1 strain oocyte co-injected with cRNAs encoding histone (red) and tubulin (green) and cyclin A1 (without florescent tag). Oocyte showed on the right was co-injected also with separase mutated to alanine on position 1121. Scale bar represents 10 μm. (**B**) CD1 oocytes were injected with histone and tubulin fused to fluorescent proteins and also with cyclin A1 (n = 16) or cyclin A1 together with separase1121 (n = 16). Polar body extrusion (PBE) and chromosome segregation were then assessed by time lapse confocal microscopy. The left chart shows frequency of PBE in each group (both groups 0% PBE). The right chart shows the frequency of chromosomes segregation in each group (cyclin A1 0%, cyclin A1 + Sep1121 50%). The data were obtained from two independent experiments. The difference between oocytes microinjected with cyclin A1 and oocytes co-injected also with separase1121 in frequency of PBE was not statistically significant (α < 0.05; P > 0.9999). The difference between oocytes microinjected with cyclin A1 and oocytes co-injected also with separase1121 in frequency of chromosome division was statistically significant (α < 0.05; **P = 0.0024). (**C**) Separase Δ oocytes were injected with histone fused to fluorescent protein and with separase 1121 (n = 23) or separase 1121 together with cyclin A1 (n = 30). PBE and chromosome segregation were assessed by time lapse confocal microscopy. The left chart shows frequency of PBE in each group (separase 1121 70% PBE, separase1121 + cyclin A1 0% PBE). The right chart shows the frequency of chromosomes segregation in each group (separase 1121 96%, separase 1121 + cyclin A1 60% of chromosomes segregated). The data were obtained from three independent experiments. The difference between separase Δ oocytes microinjected with separase 1121 and separase Δ oocytes co-injected also with cyclin A1 in frequency of PBE was statistically significant (α < 0.05; ***P < 0.0001). The difference between separase Δ oocytes microinjected with separase1121 and separase Δ oocytes co-injected also with cyclin A1 in frequency of chromosome division was statistically significant (α < 0.05; **P = 0.0033). (**D**) Separase Δ oocytes were injected with histone fused to fluorescent protein and with wild type separase (separase WT, n = 13) or wild type separase together with cyclin A1 (n = 14). The left chart shows frequency of PBE in each group (separase WT 92% PBE, Separase WT + cyclin A1 0% PBE) and the right chart the frequency of chromosome segregation in each group (separase WT 92%, separase WT + cyclin A1 7%). The data were obtained from two independent experiments. The difference between separase Δ oocytes microinjected with separase WT and separase Δ oocytes co-injected also with cyclin A1 in frequency of PBE was statistically significant (α < 0.05; ***P < 0.0001). The difference between separase Δ oocytes microinjected with separase WT and separase Δ oocytes co-injected also with cyclin A1 in frequency of chromosome division was statistically significant (α < 0.05; ***P < 0.0001).

Separase is in mouse oocytes required for conversion of bivalent chromosomes into univalents during anaphase^31^. Since the expression of cyclin A1 blocked this process (Fig. 1D), we speculated that separase might be directly affected by cyclin A1. It was shown previously that the separase activity is directly controlled not only by securin, but also by Cdk1 phosphorylation^32^ and that mutation of the residue 1121 in mouse separase, required for CDK1 control, caused genome instability^33^. We first tested whether separase, with serine to alanine mutation at position 1121 (separase 1121), could abolish the block of chromosome segregation induced by cyclin A1 expression. GV oocytes were microinjected with cRNAs encoding tagged histone H2B and tubulin and untagged cyclin A1. Half of the cells were also coinjected with cRNA encoding untagged separase 1121. Cells were subsequently monitored by time lapse confocal imaging (Fig. 4A). The results revealed that although oocytes in both groups showed no PBE (Fig. 4A,B, left chart), 50% of oocytes injected with separase 1121 divided their chromosomes (Fig. 4A,B, right chart). For this experiment we used oocytes from CD1 mice, which contained also endogenous separase. In order to analyze solely the effect of separase 1121 in cells expressing cyclin A1, we used oocytes isolated from animals with insertions of LoxP sites in both separase alleles and carrying also Cre recombinase under ZP3 promoter^31^. Because under these circumstances the endogenous separase is depleted, such oocytes (separase Δ) are unable to divide their chromosomes and to proceed into meiosis II. Separase Δ GV oocytes were injected with cRNAs of tagged histone and tubulin and untagged separase 1121. Half of them then also with untagged cyclin A1. Our results showed that 70% of separase Δ oocytes injected with separase 1121 underwent PBE (Fig. 4C, left chart), and this was completely abolished by simultaneous injection of cyclin A1. Analysis of chromosome division (Fig. 4C, right chart) showed that 96% of separase Δ oocytes injected with separase 1121 and 60% of separase Δ oocytes injected with separase 1121 and cyclin A1 divided their chromosomes. In order to test whether 1121 mutation is indeed the target of cyclin A1, we injected separase Δ oocytes with wild type separase (separase WT) in experiment similar as in Fig. 4B. Our results showed that microinjection of separase WT into separase Δ oocytes rescued in 92% cells PBE as well as chromosome segregation (Fig. 4D, left chart), but when coinjected with cyclin A1 none of the cells extruded PB and only 7% of cells segregate chromosomes (Fig. 4D, right chart). In summary, our experiments show clearly that cyclin A1 expression abolish chromosome segregation by direct inhibition of separase via serine 1121.

### Early mouse embryos are insensitive to cyclin A1 expression

**Figure 5:**
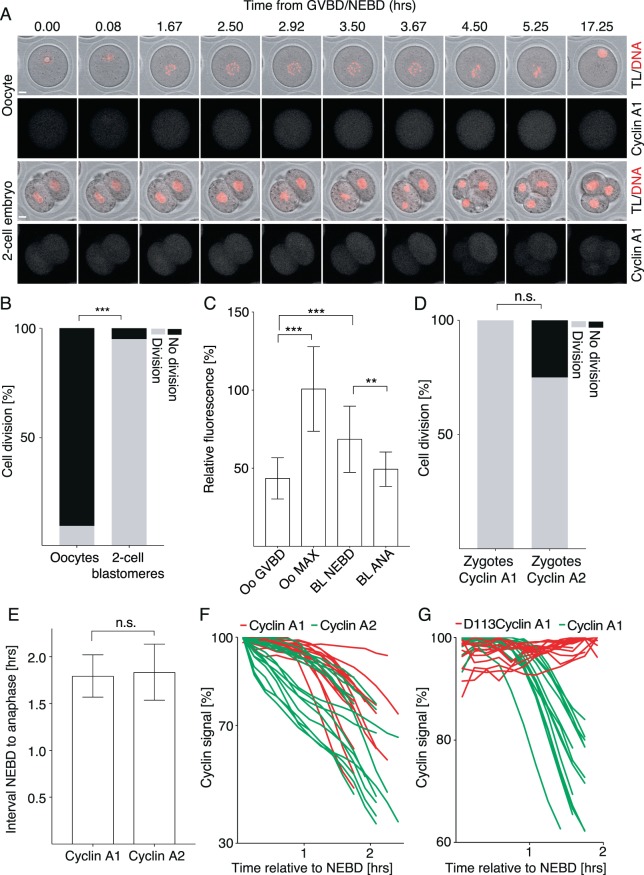
Expression of cyclin A1 does not prevent anaphase in zygotes and 2-cell embryos. (**A**) Frames from the time lapse experiment showing comparison between oocyte (upper panel) and 2-cell embryo (lower panel). Both oocytes and embryos were microinjected with cRNAs encoding histone and cyclin A1 fused to fluorescent proteins. Cell division and chromosome segregation were then assessed by time lapse confocal microscopy. Scale bar represents 10 μm. (**B**) Scoring of cell division of oocytes (n = 22) and 2-cell blastomeres (n = 42) described in panel (A). Cells which divided (oocytes 9%, embryos 95%) are indicated by grey bars and cell with no division (oocytes 91%, embryos 5%) observed during the duration of experiment are as black bars. The data were obtained from three independent experiments. The difference between oocytes and 2-cell blastomeres was statistically significant (α < 0.05; ***P < 0.0001). (**C**) Relative fluorescence signal of cyclin A1 in indicated cells and time intervals. GV oocytes – the relative fluorescence signal was measured in the first frame after GVBD (Oo GVBD, 43.24 AU) and in the frame of maximum fluorescence (Oo MAX, 102.6). 2-cell blastomeres – the relative fluorescence signal was measured in the first frame after NEBD (BL NEBD, 63.97 AU) and in the first frame of anaphase (BL ANA, 44.76). The difference between Oo GVBD and Oo MAX was statistically significant (α < 0.05; ***P < 0.0001), the difference between Oo GVBD and BL NEBD was statistically significant (α < 0.05; ***P < 0.0007) and the difference between BL NEBD and BL ANA was statistically significant (α < 0.05; **P < 0.0011). (**D**) Zygotes were microinjected with cRNAs encoding histone and either cyclin A1 (n = 13) or cyclin A2 (n = 12) fused to fluorescent proteins. Cell division was then assessed by time lapse confocal microscopy. Cells which divided (cyclin A1 100%, cyclin A2 75%) are indicated by grey bars and cell with no division (cyclin A1 0%, cyclin A2 25%) observed during the duration of experiment are as black bars. The data were obtained from two independent experiments. The difference in division between zygotes was not statistically significant (α < 0.05; P = 0.0957). (**E**) The length of the interval between disassembly of the nuclear membrane (NEBD) and anaphase was measured in cells described in panel (C). Average time interval between NEBD and anaphase was 1.79 h for cells injected with cyclin A1 cRNA and 1.83 h for cells injected with cyclin A2 cRNA. The data were obtained from two independent experiments. The difference between the length of mitosis in cells injected with cyclin A1 and cyclin A2 were not significant (α < 0.05; P = 0.7254). (**F**) The levels of fluorescence signal of cyclin A1 (red, n = 16) and cyclin A2 (green, n = 16) during mitotic division of zygotes. The signal in each cell from NEBD to anaphase is shown as individual curve. The signal was normalized to the maximum level. The data were obtained in two independent experiments. (**G**) The levels of fluorescence signal of D113cyclin A1 (red, n = 16) and cyclin A1 (green, n = 14) during mitotic division of zygotes. Individual curves for each analyzed cell. The signal in each cell from NEBD to anaphase is shown as individual curve. The signal was normalized to the maximum level. The data were obtained from two independent experiments.

Given that the presence of cyclin A1 in oocytes leads into meiosis I arrest with undivided chromosomes, we aimed to address whether the mitotic division of early embryos is also sensitive to cyclin A1 expression. For this, the GV oocytes and both blastomeres of 2-cell embryos were injected with cRNAs encoding tagged histone H2B and cyclin A1 (Fig. 5A). In order to ensure that the expression levels will be comparable, microinjection was done simultaneously with the same mixture of cRNAs and cells were matured on confocal microscope in the same dish. Our results showed that 95% of 2-cell embryos divided into 4-cell embryos, but only 9.09% of oocytes divided (Fig. 5B). The intensity of cyclin A1 signal in oocytes during GVBD and in blastomeres during NEBD was quantified (Fig. 5C). The results showed that the blastomeres expressed higher levels of cyclin A1 than oocytes during disassembly of the nuclear membrane (64 AU vs 43 AU). In contrast to oocytes however, which accumulated cyclin A1 during progression of meiosis I until the activation of APC/C (Figs. 2B and 5C), the signal of cyclin A1 in blastomeres decreased since NEBD (64 AU) until anaphase (45 AU). Since in mouse 2-cell embryos the transcription from the zygotic genome is already initiated^34^, we tested whether the insensitivity to cyclin A1 might have resulted from the gene expression at this stage and performed similar experiments with zygotes. They were injected with cRNAs of tagged histone H2B to monitor chromosome segregation and then either with cyclin A1 or A2. The results showed that 100% of zygotes expressing cyclin A1 and 75% of zygotes expressing cyclin A2 divided (Fig. 5D). And when we compared the length of the first mitosis, there was no significant difference between both groups (1.79 hours in cyclin A1 group vs 1.83 hours in cyclin A2 group) (Fig. 5E). The comparison of the individual expression levels in each cell showed that both cyclins were significantly degraded before anaphase (Fig. 5F). However, cells injected with the D113 cyclin A1, which lacks the D-box, were unable to degrade its signal before anaphase (Fig. 5G), although 100% of the cells underwent division (data not shown). This demonstrates that unlike oocytes, the embryos before and after ZGA are capable to sustain the expression of cyclin A1, without consequences for their division, chromosome segregation or for the length of mitosis.

## Discussion

In mammals, a remarkable differences between progression of male and female meiosis and the morphology of the resulting gametes are also reflected by selection of genes required during their development^35–38^. By gene knockout it was previously shown that cyclin A1 is essential for male meiosis and that it is not required for female meiosis^12^. The arrest caused by CCNA1 knockout was consistent with the expression pattern of cyclin A1 in spermatocytes, in which the protein was detectable in pachytene and diplotene stages until the spindle assembly in meiosis I^14,15^. On the other hand, the situation with expression of cyclin A1 in the oocytes is less clear. Some studies reported expression of CCNA1 mRNA and protein in GV and MII oocytes, although at a very low level^4,19^. In another study however authors failed to detect any CCNA1 transcript or cyclin A1 protein in oocytes by *in situ* hybridization, Northern blot or immunohistochemistry^20^.

Our results presented here revealed that cyclin A1 is not only dispensable for female meiosis, but in fact its presence in oocytes in undesired and would have severe consequences for metaphase to anaphase transition. Our findings were supported by a recent report, which was published after the submission of our manuscript, in which authors show similar effect of cyclin A1 on progression of meiosis^39^. Our results further showed that cyclin A1 mRNA is absent in oocytes or expressed at very low levels. Quantification of endogenous cyclin A1 mRNA, using sensitive qPCR with TaqMan probes, revealed that the expression of this transcript relative to cyclin A2 and cyclin B1 transcripts is in oocytes dramatically low. The ratio of expression of cyclin A1 to cyclin A2 and cyclin B1 transcripts was in spermatocytes, in which the cyclin A1 is essential, 13–18 times lower, whereas in oocytes this ratio was 119–162 times lower.

It seems that the major problem, caused by the presence of cyclin A1 in oocytes, is the inability of APC/C and proteasome to degrade this protein and its associated kinase activity before anaphase. Experiments comparing the expression of cyclins A1 and A2 side by side seems to support this. The retention of cyclin A1 is perhaps not initially dependent on kinase activity, since both cyclins likely form complexes with Cdk2/1 kinases and cells arrested only after microinjection of cyclin A1. Also, the experiments with the 113 cyclin A1 showed that the targeting of this fragment containing D-box but not the Cdk binding domain, is not efficient. The signal of this fragment during anaphase was higher than at the GVBD, which was not observed in the case of securin or cyclin B1 fragment. Additionally, the expression of this fragment also affected dynamics of targeting of both securin and cyclin B1. It is therefore conceivable that the N-terminal portion of cyclin A1 is interfering with APC/C – proteasome axis in oocytes.

Inefficient targeting by APC/C and proteasome, leading into retention of cyclin A1 during anaphase, has two major impacts on female meiosis. One consequence is the blockage of the polar body extrusion. This was in fact manifested in all cells expressing the C-terminal fragment and in almost all cells expressing full length cyclin A1. And it seems that even a very low amount of cyclin A1 is sufficient to block the PBE^40^. Another consequence of having cyclin A1 is the block of conversion of bivalent chromosomes into univalents, which is required for homologous chromosome segregation and for transition into meiosis II^41^. It was shown that in oocytes this process requires separase since the prophase pathway is not active in these cells^31^. Both phenotypes - the absence of PBE and the block of chromosome segregation after cyclin A1 expression are perhaps independent, since the experiments with separase 1121 showed that this mutation can restore, to certain extent, the resolution of bivalents, but not the PBE. This is not surprising as it was shown previously that oocytes expressing separase without proteolytic activity or with cohesin subunit Rec8 resistant to separase, are able to extrude PB^31,42^. Experiments with mutated separase 1121 nevertheless clearly showed that the residual amount of cyclin A1, which remains in the cell at the time of anaphase entry, is sufficient to inhibit the activity of separase and to prevent chromosome segregation. This is in contrast to the recently published results claiming that separase is prematurely activated during cyclin A1 or stable cyclin A1 overexpression^39^. Our data also showed a discrepancy between overexpression of full-length cyclin A1 and the stable version of this protein, namely in the different ability of both to block the separase activity. In case of the stable cyclin A1, only the higher expression levels were able to prevent chromosome segregation. Although it was shown that the deletion of 90 N-terminal amino acids does not prevent association of cyclin B with separase and the inhibition of separase activity^43^, in our case the N terminal deletion of cyclin A1 was longer and we also cannot exclude a possibility that there are other motifs in the N-terminal portion of cyclin A1, which would explain the lower efficiency of D113 fragment towards separase.

Surprisingly, our experiments showed that the zygotes and the 2-cell embryonic blastomeres, which are just two, respectively three divisions far from MI oocytes, tolerate expression of cyclin A1 with no obvious problem or delay in division. Our data showed that the embryos are not only able to reduce cyclin A1 levels before anaphase, but in case of stable cyclin A1 they enter anaphase with a relatively high levels of this protein. Especially the later result is very surprising and the only explanation in our opinion could be that the stable cyclin A1 is not coupled in the embryos with the kinase or that the complex is inactive. It was shown that whereas the Cdk1 is essential for mouse embryo development^44^, the Cdk2 is not essential and embryos develop normally in its absence^44,45^. It is therefore conceivable that the Cdk2, as a main partner of A type cyclins, is absent in zygotes and thus the stable cyclin A1 is tolerated in this developmental stage. However further experimental work is necessary to elucidate if the cyclin A1 is able to form a complex with Cdk2 kinase in zygotes and 2-cell embryos and whether this complex is active.

Overall our data showed that the cyclin A1 expression discriminates between the male and female mammalian meiosis. From the literature it is clear that this molecule is required for male meiosis^12^. However, so far, we have no information about the function of this protein in sperm other than that it might required for the initiation of meiosis I via CDC25 activation^18^. It is conceivable that during the resumption of meiosis, oocytes require different activator of this phosphatase, mainly because the initiation and completion of prophase in meiosis I is in mammalian oocytes uncoupled nd separated by a substantial time interval. Our experiments here and also currently published results^39^ clearly showed that meiosis I progression in oocytes is incompatible with cyclin A1 expression. This gene must be therefore strictly controlled in both male and female germ cells, ensuring sufficient expression in the first and suppression of expression in the later.

## Methods

### Animals

BDF1 and CD1 mouse strains were purchased from Anlab (Czech Republic) or Animal Breeding and Experimental Facility (Masaryk University, Czech Republic). CD1/BDF1 female mice were obtained from crossing between CD1 female and BDF1 male. Separase flox mice generation was previously described^31^. For embryos isolation the females were stimulated with Pregnant Mare Serum Gonadotropin (PMSG, 5 IU, Merck) followed 44–48 hours by stimulation with Human Chorionic Gonadotropin (hCG, 5 IU, Merck) prior the mating. All animal work was conducted in accordance with Act No 246/1992 Coll., on the protection of animals against cruelty under the supervision of the Central Commission for Animal Welfare, approval ID 51/2015.

### Oocytes and embryos isolation and microinjection

Ovaries were excised and transferred into M2 medium (Merck) containing 0.1 mM 3-isobutyl-1-methylxanthine (IBMX, Merck) to keep immature oocytes. Germinal vesicle (GV) stage oocytes were isolated and subsequently cultured at 37 °C in 5% CO_2_ in M16 medium (Merck) with IBMX, covered with mineral oil (Merck or YBUX) for at least 1 hour prior to microinjection. Zygotes and 2-cell embryos were isolated from oviducts 18–21 and 45–47 hours post hCG stimulation in M2 medium (Merck) and subsequently cultured in KSOM + AA (Caisson Laboratories) covered with mineral oil (Merck or YBUX) at 37 °C, 5% CO_2_. For removing of the cumulus cells was used 0.05% hyaluronidase from bovine testes (Merck). Microinjection was performed in M2 medium with (oocytes) or without (embryos) IBMX inhibitor using I10 Narishige microinjector on a Leica DM IL inverted microscope. After microinjection were oocytes (M16 medium + IBMX, Merck) and embryos (KSOM + AA, Caisson Laboratories) cultured for cRNA expression for 1 to 3 hours prior to live cell imaging assay (37 °C, 5% CO_2_).

### DNA vectors and *in vitro* transcription

Following ORFs were sub-cloned into modified pBluescript RN3 vector – histone 2B (H2B), beta tubulin, securin, cyclin A1, D113cyclin A1 (deletion of first 339 nucleotides of mouse cyclin A1 ORF – cyclin A1 without D-box), 113cyclin A1 (first 339 nucleotides of mouse cyclin A1 ORF – D-box fragment of cyclin A1 itself), cyclin A2, cyclin B1, 90cyclin B1 (first 270 nucleotides of mouse Ccnb1 ORF), separase, separase1121 (ORF encoding separase was modified by QuikChange II Site-Directed Mutagenesis Kit (Agilent Technologies) at Serine position 1121 to Alanine^33^). Some ORF were fused to various fluorescence proteins, such are CFP, EGFP, YFP and mCherry. cRNAs for microinjection were prepared by *in vitro* transcription from T3 promoter (mMESSAGE mMACHINE kit, Thermo Fisher Scientific) with subsequent polyadenylation (Poly(A)Tailing kit, Thermo Fisher Scientific).

### Live cell imaging

Live cell imaging assay was performed using Leica SP5 and Olympus FluoView 3000 confocal microscopes, both equipped with EMBL incubator. Oocytes and embryos were scanned every 5 or 10 minutes with HCX PL APO 20×/0.7 IMM CORR λBL, HCX PL APO 40×/1.1 water CS, UPLSAPO 30x S/1.05 SILICONE objectives. Wavelengths 458 or 448, 488, 514, 561 or 594 nm were used for excitation and Laica HyDs or Olympus GaAsP were used for the detection of CFP, EGFP, Venus and mCherry fluorescent proteins. Obtained data were processed and analyzed using ImageJ (http://rsb.info.nih.gov/ij/), Leica LAS AF (http://www.leica-microsystems.com) or Imaris (www.bitplane.com) software.

### Immunostaining

Non-injected or microinjected oocytes were subsequently maturated and then treated with 5 μM dimethylenastron (Merck), fixed in 2% paraformaldehyde (Merck) for 20 minutes, permeabilized with 0.1% Triton X-100 (Merck) for 15 minutes and blocked (0.1% BSA) for 1 hour. The human antibody against centromere (1:500 dilution, Immunovision) and Alexa Fluor 647 goat anti-human (1:500 dilution, ThermoFisher Scientific) were used. Cells were mounted in vectashield with DAPI (Vector Laboratories). Images were acquired using Leica SP5 confocal microscope equipped with HCX PL APO 63×/1.4 oil objective, 405 and 633 nm wavelengths were used for excitation and PMT detectors were used for detection of the signal.

### Preparation of spermatogenic cell suspension

Testes from 15 to 18 days old CD1 males were excised and tunica albuginea was removed in DPBS. Cell suspensions was prepared from partially dissociated tubules. Cells from the sediment were washed twice in DPBS and then incubated for 4–6 minutes in 0.1–0.25% trypsin and 20 μg/ml DNase in dissolved in DBPBS. During this time the tubules were dispersed by pipetting with a Pasteur pipette. After approximately 5 minutes, the integrity of the tubules was visually checked and if they were dispersed, Soybean trypsin inhibitor (SBTI) was added to the final concentration of 0.5 mg/ml. Tissue aggregates were removed by filtration using 50 μm nylon mesh and then the cell suspension was washed three times with DPBS containing 0.5% BSA. The presence of spermatocytes, the large cells with characteristic nuclei, was assessed by microscopy their morphology was examined after staining with Hoechst 33342 (10 μg/ml, Sigma-Aldrich).

### Total RNA isolation, reverse transcription and qPCR

Extraction of total RNA from oocytes (50 GV oocytes per sample) or spermatogenic cell suspension was performed by RNeasy Plus micro kit (QIAGEN) following to manufacturer’s instructions. Final elution was done by dH2O in amount of 50 µl for oocyte and 50 µL per spermatocyte samples. Quality and quantity control of isolated samples was evaluated by Bioanalyzer 2100 with RNA 6000 Pico kit (Agilent). Reverse transcription to complementary DNA was made by qPCRBIO cDNA synthesis kit (PCR BIOSYSTEMS) using oligo(dT) (ThermoFisher Scientific) and random hexamer primers (ThermoFisher Scientific). Program was set up on 30 minutes at 42  °C. Samples of oocytes and spermatogenic cell were stored at −20  °C. Quantitative PCR was performed by CFX96 Real time system (Biorad) using TaqMan Gene Expression Master Mix XS (Applied Biosystems) recommended by the manufacturer. Final reaction volume of quantitative PCR was 20 µL. The concentration of primers and Taqman probes with FAM reporter dye was 5 nM. Predesign primer sets (Applied Biosystems) were used against target: Mm00432337m1 (cyclin A1), Mm00438063m1 (cyclin A2), Mm03053893gH (cyclin B1). Quantitative PCR reactions: 50  °C/2 minutes, 95  °C /10 minutes, followed by 40 cycles of 95 °C/15 second, 50  °C /20 second and 60  °C /60 second. For quantitative PCR reactions were prepared in triplicates per run. To quantify gene expression level, cycle threshold (CT) value was determined and the fold change of gene expression was calculated using 2^−ΔΔCT^ method. Expression of each cRNA was normalized to that of cyclin A2. Presented data are obtained from minimally three biological replicates of oocytes and spermatogenic samples (Table 1).

**Table 1:** Expression of cyclin A1 transcript in comparison to cyclin A2 and cyclin B1 transcripts is dramatically lower in oocytes than in spermatocytes.

Average Ct for indicated transcripts			
	*cyclin A1*	*cyclin A2*	*cyclin B1*
GV oocyte	38.36 ± 0.89	27.47 ± 0.63	25.63 ± 0,36
Spermatocyte	30.38 ± 1.16	26.78 ± 0.53	26.08 ± 0.89
	ΔCt for indicated transcripts	ΔCt (cyclin A1 – cyclin A2)	ΔCt (cyclin A1 – cyclin B1)
	GV oocyte	10.89	12.73
	Spermatocyte	3.60	4.30

Expression levels of cyclin A1, cyclin A2 and cyclin B1 mRNA were analyzed in GV oocytes and spermatocytes using qPCR and TaqMan probes. The threshold cycle (Ct) for each transcript is indicated. The relative expression of *cyclin A1* to *cyclin A2* and *cyclin B1* was calculated as a difference between the Cts for each transcript.

### Statistics

Statistical analysis of the data was done by performing t-test, Mann Whitney test, Chi-square test and Fisher’s test in Prism software, version 7.0a for Mac (GraphPad Software, San Diego, CA, USA; www. graphpad.com).

## Supplementary information

Supplementary information.
