# Supplementary material for: Cyclin A1 in Oocytes Prevents Chromosome Segregation And Anaphase Entry
Source: Sci Rep. 2020 May 4;10:7455. doi: 10.1038/s41598-020-64418-1 (PMC7198627; doi:10.1038/s41598-020-64418-1)

# CYCLIN A1 IN OOCYTES PREVENTS CHROMOSOME SEGREGATION AND ANAPHASE ENTRY

Lenka Radonova[1,2], Tereza Pauerova[1,2], Denisa Jansova[2], Jitka Danadova[1], Michal Skultety[1,2], Michal Kubelka[2] and Martin Anger[1,2]*

[1]Central European Institute of Technology, Department of Genetics and Reproduction, Veterinary Research Institute, Brno, Czech Republic
[2]Institute of Animal Physiology and Genetics, Czech Academy of Sciences, Libechov, Czech Republic

* corresponding author, email: anger@vri.cz

A

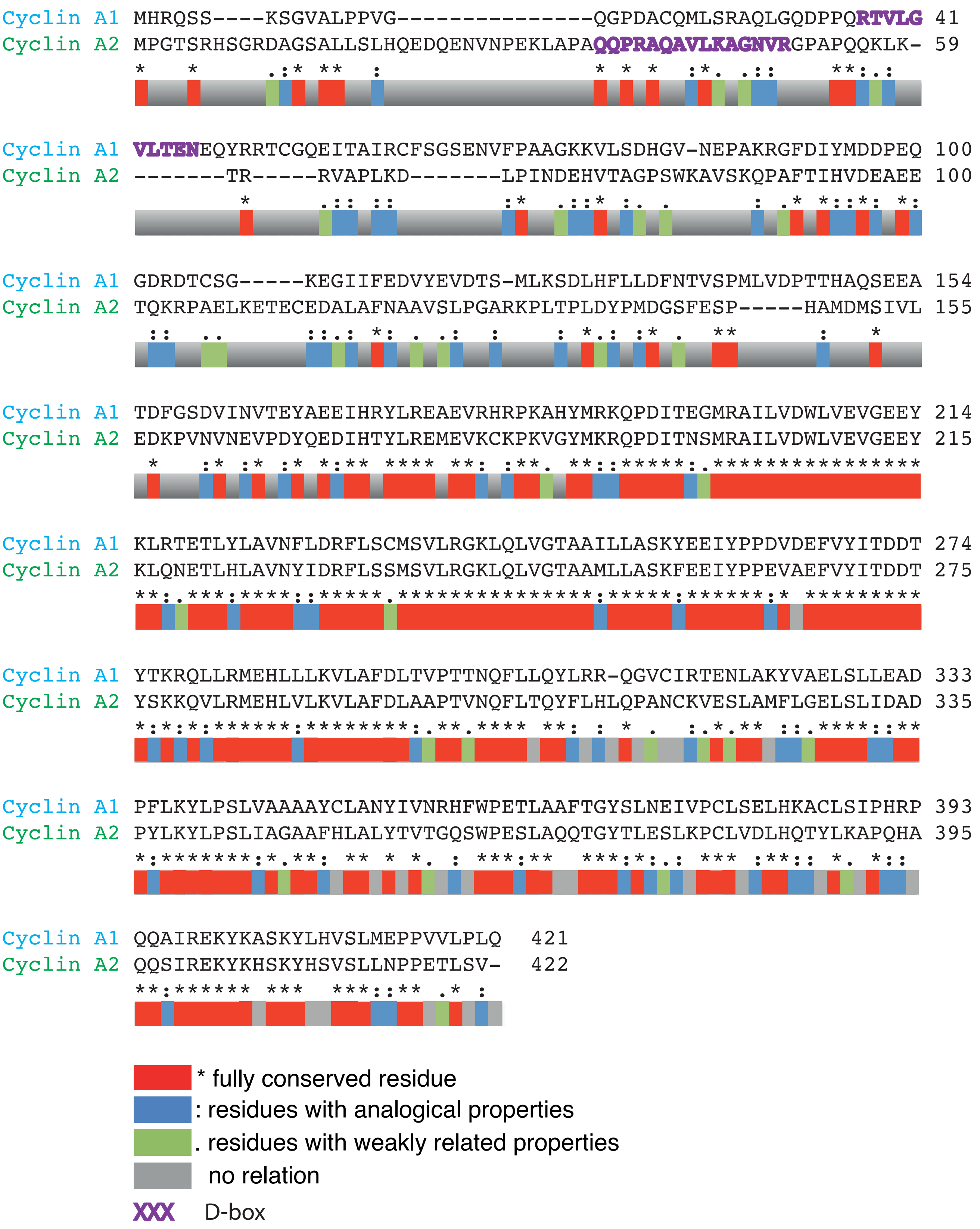

Supplement: Supplementary file 1 — Supplementary information. [file 41598_2020_64418_MOESM1_ESM.pdf]
